# Optimization of mannanase expression in *Aspergillus niger* for enhanced production of mannan oligosaccharides

**DOI:** 10.3389/fbioe.2025.1716075

**Published:** 2026-01-30

**Authors:** Hui Zhang, Huakai Tang, Tingting Ma, Xiaofei Ma, Yuwei Kou, Bing Wang, Jiahuan He, Jie Li

**Affiliations:** School of Life Sciences at Northeast Agricultural University, Harbin, China

**Keywords:** *Aspergillus niger*, gene overexpression, mannan oligosaccharides, mannanase, signal peptide, sorting 10, vacuolar protein

## Abstract

Mannan oligosaccharides (MOS) are high-value prebiotics widely applied in the food, feed, and pharmaceutical industries. Mannanase, as the key rate-limiting enzyme in the biosynthesis of MOS, directly determines MOS yield and production cost based on its expression level. However, current industrial enzyme sources commonly exhibit low expression levels, poor secretion efficiency, and inadequate stability, severely limiting the reliable production of high-performance enzyme preparations. To overcome this core bottleneck, this study employed *Aspergillus niger*-an established industrial host-as the chassis organism and systematically improved the expression level and extracellular secretion efficiency of mannanase through integrated multi-dimensional strategies, including gene copy number regulation, secretion pathway optimization, and signal peptide modification. This project aims to provide efficient enzyme resources and robust technical support for the cost-effective and scalable biosynthesis of MOS. Specifically, Doubling the gene copy number enhanced *man* transcript levels 92.00% and extracellular mannanase activity by 66.25%, while tripling the copy number induced the unfolded protein response (UPR) and impaired growth. Deletion of AsAA relieved secretion stress, leading to a 10.11% increase in transcription and a 175.64% improvement in enzyme activity. Similarly, deleting *Vps10* resulted in a 24.48% increase in transcription and a 30.08% increase in enzyme activity, likely by reducing degradation of folded protein. Replacement of the SglaA signal peptide with SpepB resulted in a 7.29% increase in transcript abundance and a 15.41% increase in enzyme activity. RT-qPCR analysis confirmed upregulation of several UPR-related genes (*hacA*, *bipA*, and *pdiA*) in most strains, consistent with ER stress under high expression burdens. The recombinant mannanase demonstrated high hydrolytic activity against konjac powder, yielding a total MOS content of 94.47% in the hydrolysis products, which highlights its significant potential for the efficient production of functional oligosaccharides. This study not only provides essential technical support for the industrial-scale synthesis of MOS, but also establishes a scalable engineering framework for the efficient heterologous expression of industrial enzymes.

## 1 Introduction

Mannan oligosaccharides (MOS) are a class of functional oligosaccharides consisting of 2–10 mannose residues connected by glycosidic bonds. They exhibit multiple biological activities, including the maintenance of intestinal health, modulation of immune function, and the potential to serve as alternatives to antibiotics. MOS are widely applied in livestock and poultry farming, aquaculture, infant formula, and functional food and dietary supplement industries. The animal feed sector accounts for approximately 70% of total demand, while the human nutrition sector, including functional foods and health products, represents about 30% (Kamal et al., 2025). Currently, the industrial-scale production of MOS is primarily based on enzymatic synthesis, while natural extraction serves as a supplementary method. β-1,4-mannanase can specifically recognize and cleave the β-1,4-glycosidic bonds of large molecular mannan derived from plant raw materials such as konjac flour and guar gum, hydrolyzing them into target oligosaccharide fragments, thereby achieving an efficient conversion from natural polysaccharide raw materials to high-purity MOS (Liao et al., 2025; Faustino et al., 2021). In 2023, the global production capacity of MOS was estimated at 80,000–100,000 metric tons per year, corresponding to an annual demand for β-mannanase of 12,000–15,000 metric tons, based on an enzyme activity of 10,000 U/g. It is projected that by 2030, the global annual production capacity of MOS will reach 180,000–200,000 metric tons, with a corresponding increase in β-mannanase demand to 30,000–35,000 metric tons per year. However, in current industrial production processes, the cost of β-mannanase constitutes 30%–40% of the total manufacturing cost of MOS, representing a critical bottleneck that limits its large-scale application. Therefore, improving the expression levels and catalytic efficiency of β-mannanase through genetic engineering has emerged as a key strategy for overcoming cost barriers and enabling the large-scale industrial application of MOS.

β-Mannanases are endo-glycosidases or glycosyl hydrolases that hydrolyze the beta-1,4-D-glucan bonds of polysaccharides such as glucans and glucomannans to produce glucose or glucan oligosaccharides (Fülöp, 2024). Mannanases are popular in the food and feed industries to enhance digestibility and nutrient utilization. For example, supplementing animal feed with *β*-mannanase stimulates digestive enzyme secretion, improves intestinal microflora, and reduces feed costs (Balasubramanian et al., 2018; Dawood and Ma, 2020; Peng et al., 2023). This enzyme also serves as a viable alternative to antibiotics in livestock and poultry for managing infections and reducing pollution, and also has applications in food processing (Prima et al., 2017; Zhao et al., 2019), oil drilling (Comfort et al., 2004), papermaking (Yang et al., 2017), and textiles (Palackal et al., 2007).

The production of industrial-grade mannanase is predominantly based on expression systems utilizing *Pichia pastoris* and *filamentous fungi*, whereas bacterial hosts are less commonly employed. When different species are employed as microbial cell factories in fermentation processes, they demonstrate distinct engineering advantages stemming from inherent variations in metabolic pathways, protein secretion systems, and post-translational modification capacities. Heterologous expression of the target enzyme enables a direct enhancement of both expression yield and catalytic activity through utilization of the host species’ specialized protein secretion pathways and post-translational modification systems (Wakai et al., 2017; Li et al., 2022). This strategy enables the targeted selection of expression systems that fulfill industrial requirements, such as enhanced biosafety and host strains with established regulatory acceptance, thereby ensuring alignment with the compliance standards of large-scale manufacturing (Schuster et al., 2002). Furthermore, substantial inter-species variations exist in host adaptability to culture medium composition and tolerance to key fermentation process parameters. The selection of host organisms with minimal nutritional demands and high environmental resilience enables both reduction in raw material costs and simplified process control, thereby achieving cost-effective and efficient fermentation processes that enhance the economic viability and environmental sustainability of industrial production (Toghueo et al., 2018; Ntana et al., 2020).

Currently, approximately 50% of commercially available mannanase is derived from *A. niger*, widely considered the best system for producing eukaryotic proteins due to its safety and long history of use in large-scale fermentation (Lübeck and Lübeck, 2022; Li et al., 2023). *A. niger* is capable of protein yields of up to 40 g·L^−1^ under industrial conditions, and can perform glycosylation and other post-translational modifications, making this species ideal for an array of applications in the food and fermentation industries (Madhavan et al., 2022). However, the choice of expression elements, together with the stringent intracellular quality control of secretory proteins, severely limits the efficient production of heterologous protein in *A. niger*. These constraints hinder high-level secretion and large-scale industrial application of many proteins with significantly potential. The *Aspergillus niger* strain employed in this study is an industrial glucoamylase-producing strain and a morphological mutant of *Aspergillus niger*. It exhibits favorable traits including the inability to produce spores, short and robust mycelial morphology, low fermentation broth viscosity, high enzyme production capacity, reduced diversity of secreted proteins, low protease activity, and tolerance to high osmotic pressure, rendering it well-suited for high-density submerged liquid fermentation. The primary high-abundance secretory proteins in this strain include glucoamylase (GlaA), α-amylase (AmyA1 and AmyA2), and acid-stable α-amylase (AsAA). To investigate whether downregulation of background protein expression can alleviate the competitive burden imposed on the target protein during the secretion process, a genetically well-characterized and technically tractable locus was selected for genetic intervention. The GlaA locus is located within a transcriptionally active genomic region and serves as a critical site for the targeted integration of heterologous proteins via homologous recombination. Given that its transcriptional activity is essential for driving high-level expression of recombinant proteins, this locus must be preserved and is therefore unsuitable as a target for gene knockout. The AmyA locus harbors two functional paralogous genes (AmyA1 and AmyA2), and genetic editing of this locus necessitates simultaneous modification of both copies, posing significant technical challenges and potentially disrupting host metabolic homeostasis. In contrast, AsAA is a single-copy gene with a well-defined and simple genomic locus. Its encoded product constitutes a significant proportion of the total extracellular protein fraction and is therefore representative. Gene knockout of AsAA effectively reduces the secretory burden imposed by non-target proteins while circumventing the technical challenges associated with multi-gene redundancy. Based on the aforementioned analysis, this study selected the AsAA gene as the genetic target and established a pyrG-based bidirectional selection system to enable precise and efficient genome editing. The mechanism of this system is dependent on orotate-5′-phosphate decarboxylase, encoded by the pyrG gene, a critical enzyme in the uracil nucleotide biosynthesis pathway. During positive selection, wild-type strains possessing a functional pyrG gene convert 5-fluoroorotic acid (5-FOA) present in the culture medium into the toxic metabolite 5-fluorouridine monophosphate (5-FUMP), resulting in cell death. In contrast, the pyrG deletion mutant is incapable of catalyzing this conversion and therefore survives on 5-FOA-containing medium, enabling the successful isolation of the stable pyrG-deficient strain TH-2 (Du et al., 2014). During negative selection, replacement of the AsAA coding region with the pyrG marker gene resulted in successful recombinants regaining pyrG function, enabling autonomous synthesis of essential nucleotides and normal growth on uracil-deficient medium. In contrast, non-integrated strains failed to synthesize uracil and exhibited growth inhibition, thereby allowing efficient identification of the AsAA knockout strain TH-2 (asAA::pyrG) (Nguyen et al., 2016). This bidirectional screening strategy not only enabled effective elimination of the AsAA background protein but also fully leveraged the high efficiency and scarless recombination capabilities of the pyrG system in gene editing, thereby establishing a robust genetic platform for investigating the relationship between background protein expression levels and target protein secretion capacity.

In eukaryotic cells, nearly one-third of newly-synthesized proteins are degraded via the secretory pathway (Schubert et al., 2000; Zhou et al., 2018; Schwabl and Teis, 2022). To enhance the yield of recombinant *β*-mannanase, it is crucial to prevent expressed proteins from entering this intracellular degradation pathway. Several quality control mechanisms ensure the transport of only appropriately folded and modified proteins from the endoplasmic reticulum (ER) to the Golgi apparatus, including the unfolded protein response (UPR) pathway, the ER-associated degradation (ERAD) pathway, the Golgi quality control (GQC) pathway, and the ER autophagy (ER-phage) pathway (Fiedler et al., 2018; Sun and Brodsky, 2019; Wang et al., 2020; Li et al., 2022). These processes evolved to prevent the accumulation of misfolded proteins within the ER, which leads to stress and disrupts normal cellular function. When proteins are overexpressed, cells utilize these pathways to degrade misfolded proteins, facilitate proper protein folding and secretion, and modulate protein synthesis, thereby maintaining proteostasis and ensuring normal cellular function (Carvalho et al., 2011; Wu et al., 2017). However, from the standpoint of secreted protein production, these same quality control mechanisms restrict the cell’s capacity for efficient secretion, thereby representing a major bottleneck in the secretory expression of recombinant proteins.

Vacuolar protein sorting 10 (Vps10) receptors direct proteins secreted by the ER to the lysosome/vacuole and serve as crucial regulators of the GQC pathway. Vps10 is a Type I transmembrane receptor protein containing an N-terminal Vps10-D domain, a primary ligand-binding site, and two small C-terminal cysteine-rich domains (10CCa and 10CCb) (Mitok et al., 2022). Predominantly localized to the Golgi compartment, Vps10 facilitates the sorting of lysosomal hydrolases (e.g., carboxypeptidase) and subsequently transports them to the vacuole. This process is similar to the mammalian lysosomal protein sorting mechanism. However, Vps10 mediates the transport of soluble proteases to the vacuole by recognizing peptide, rather than carbohydrate, signals on the unnecessary proteins. In this way, soluble proteins containing peptide sequences similar to vacuolar proteases are recognized by Vps10 and transported to the vacuole for degradation, ultimately reducing protein expression levels. Vps10 mediates the targeted degradation of misfolded and recombinant proteins via the Golgi-lysosomal pathway (Huang et al., 2018). Studies have demonstrated that deletion of Vps10 significantly enhances the extracellular secretion efficiency of both endogenous and recombinant proteins (Yoon et al., 2010; Luo et al., 2022). Given that mannanase in this study is classified as a soluble endogenous protein, its secretion pathway may be subject to regulation by Vps10. Therefore, this study aims to evaluate the feasibility of enhancing extracellular mannanase secretion by deleting the Vps10 gene, thereby leveraging the regulatory mechanism of the Vps10-mediated degradation pathway to overcome secretion bottlenecks and improve the level of extracellular secretory expression.

Signal peptides are critical determinants of target protein secretion and expression efficiency, as their functional properties directly govern the rate of transmembrane translocation, the extent of extracellular secretion, and the fidelity of protein folding (Wang et al., 2015; Nieuwkoop et al., 2020; Bhadra and Helms, 2021; Smets et al., 2022). In our previous work, the SglaA signal peptide derived from the glucoamylase gene of *Aspergillus niger* was employed to direct the expression of target proteins, resulting in a significant enhancement of extracellular secretion (Xu et al., 2018), thereby demonstrating its high efficiency as a secretion signal in the homologous fungal host system. Sequence analysis reveals significant differences between SpepB and SglaA in key functional domains: the hydrophobic core region (H-region) of SpepB consists of a continuous stretch of hydrophobic amino acids (Val-Val-Phe-Ala-Ala-Leu), uninterrupted by polar residues, indicating more uniform and stable hydrophobicity that enhances its binding affinity for the signal recognition particle (SRP) and improves endoplasmic reticulum (ER) targeting efficiency. The C-terminus of the SpepB signal peptide terminates with alanine (Ala), conforming to the conserved cleavage site preference of fungal signal peptidase. This structural feature is associated with a high predicted cleavage efficiency, thereby promoting complete removal of the signal peptide and minimizing potential structural or functional interference caused by residual peptide fragments on the target protein. Moreover, SpepB lacks cysteine (Cys) residues, thereby avoiding the formation of non-specific disulfide bonds and reducing the likelihood of protein misfolding and endoplasmic reticulum (ER) retention. In contrast, SglaA contains a single Cys residue, which may disrupt the molecular chaperone-mediated folding process and compromise proper conformational maturation of the target protein. Although SglaA has demonstrated favorable secretion performance, SpepB displays superior sequence features with respect to transport efficiency, cleavage accuracy, and folding compatibility, thereby more fully satisfying the integrated functional demands of signal peptides within the fungal secretory pathway-particularly in the coordinated regulation of transmembrane translocation, efficient post-translational processing, and proper protein folding. Therefore, this study employs the substitution of the SpepB signal peptide to explore its potential to overcome the “secretion ceiling” associated with SglaA, thereby establishing a more optimized strategy for achieving efficient extracellular expression of the target protein.

MOS exhibit significant potential for application in various fields, including food science, nutritional supplements, and animal feed. The expression level and catalytic activity of mannanase directly influence the yield of MOS. Therefore, this study aimed to enhance mannanase expression by increasing the copy number of the gene expression cassette, deleting the high-secretion background protein AsAA, disrupting the vacuolar protein sorting receptor-encoding gene Vps10 involved in the degradation pathway, and systematically comparing the efficacy of two signal peptides (SglaA and SpepB). Furthermore, the impact of these genetic optimization strategies on mannanase production was comprehensively evaluated. The hydrolysis conditions and resulting products were systematically analyzed to enhance the preparation efficiency of MOS and to establish a solid foundation for their large-scale and high-efficiency production.

## 2 Materials and methods

### 2.1 Strains and culture conditions

*A*. *niger* ‘TH-2’ was obtained from Zhaodong Richeng Enzyme Preparation Co., Ltd. (Zhaodong, China). The *A. niger* ‘TH-2 (ΔasAA::pyrG)’ and the *A. niger* ‘TH-2 (Vps::pyrG)’ were both constructed in our laboratory. *Agrobacterium tumefaciens* ‘AGL1’ was maintained in our laboratory. The cloning vector pMD19-T (simple), T4 DNA ligase, and restriction endonucleases (*Nhe* I, *Xba* I, and *Hind* III) were all purchased from Takara Bio (Dalian) Co., Ltd. (Dalian, China). The Ultra-Pure RNA Extraction Kit (including DNase I), HiFiScript gDNA Removal cDNA Synthesis Kit, UltraSYBR One-Step RT-qPCR Kit, Plant Genomic DNA Extraction Kit, Agarose Gel DNA Recovery Kit, and High-Purity Plasmid Mini Kit were all purchased from Beijing Kangwei Century Biotechnology Co., Ltd. (Beijing, China). Rifamycin (Rif), kanamycin (Km), cefalexin, acetylsyringone (AS), ampicillin (Amp), hygromycin B (HygB), 5-fluoroorotic acid (5-FOA), and uridine were purchased from Beijing Solarbio Science and Technology Co., Ltd. (Beijing, China). *Escherichia coli* ‘DH5α’ and *Agrobacterium tumefaciens* ‘AGL1’ were utilized for DNA manipulation and multi-copy plasmid construction, respectively. Following transformation, *Escherichia coli* cells harboring the recombinant plasmid were shake-cultured at 37 °C and 200 r·min^−1^ in Luria-Bertani (LB) medium (1 g·L^−1^ yeast extract, 10 g·L^−1^ peptone, and 10 g·L^−1^ NaCl; pH 7.0). *A. tumefaciens* was cultured in yeast extract beef (YEB) medium (1 g·L^−1^ yeast extract, 5 g·L^−1^ peptone, 5 g·L^−1^ beef extract, 5 g·L^−1^ sucrose, and 0.493 g·L^−1^ MgSO_4_·7H_2_O; pH 6.5) at 28 °C. *A. niger* ‘TH-2’ was cultured in potato dextrose agar (PDA) (200 g·L^−1^ potato extract, 20 g·L^−1^ glucose, 3 g·L^−1^ KH_2_PO_4_, and 1.5 g·L^−1^ MgSO_4_·7H_2_O; pH 7.0) at 30 °C. Recombinant mannanase-expressing *A. niger* was cultured in shake flasks at 30 °C using industrial fermentation medium (100 g·L^−1^ glucose, 20 mL·L^−1^ corn steep liquor, and 20 g·L^−1^ soybean powder; pH 5.5–6.0). To prepare solid media, each medium was supplemented with 20 g·L^−1^ of agar and pressure sterilized at 121 °C for 30 min. Each culture was initiated with a 10% (v/v) inoculum of the strain and incubated at 200 r·min^−1^ under precisely controlled environmental conditions.

### 2.2 Cloning of mannanase gene and construction of expression vectors

**FIGURE 1:**
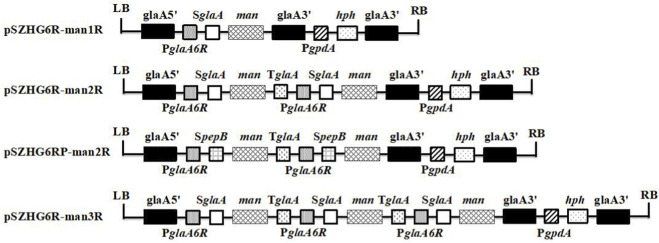
Schematic representation of the mannanase expression cassettes constructed in this study. *glaA5’* (1,200 bp) and *glaA3’* (1,100 bp) represent the homologous arms, with *glaA3’* (1,200 bp) containing a transcriptional terminator. *hph* (900 bp) is the selection marker, driven by the *PgpdA* (2,129 bp) promoter. P*glaA6R* is a strong six-copy promoter for *glaA*, and T*glaA* (800 bp) represents the terminator sequence. LB and RB indicate the left and right 25 bp flanking repeats of the T-DNA region. Plasmid constructs include: pSZHG6R-man1R (one-copy cassette), pSZHG6R-man2R (two-copy cassette), pSZHG6RP-man2R (two-copy cassette with *SpepB* signal peptide), and pSZHG6R-man3R (three-copy cassette).

The 1,200 bp mannanase (*man*) coding sequence (CDS) was amplified from *A. niger* ‘TH-2’ genomic DNA using the ManMC-FW and ManMC-RW primers (Table 1). Next, the six-copy strong promoter P*glaA6R* and the high-expression signal peptide S*glaA* were integrated into the vector to obtain the PglaA6RS construct. Following *Nhe* I/*Hind* III digestion, the T-man vector and the T-PglaA6RS vector were ligated with T4 DNA ligase to obtain the one-copy expression vector T-PglaA6RS-man. The T-PglaA6RS-man and T-T*glaA* vectors were subsequently digested with *Bgl* II/*Hind* III and ligated to obtain the one-copy T-PglaA6RS-man-T*glaA* expression vector with *glaA* terminator. This vector was double-digested with *Nhe* I/*Hind* III and ligated to the double-digested (*Xba* I/*Hind* III) T-PglaA6RS-man vector to obtain the two-copy T-PglaA6RS-man-T*glaA*-PglaA6RS-man expression vector. Finally, the one-copy (T-PglaA6RS-man) and two-copy (T-PglaA6RS-man-TglaA-PglaA6RS-man) expression vectors were digested with *Xba* I/*Hind* III and the PglaARD vector was digested with *Nhe* I/*Hind* III to obtain the one-copy and two-copy *A. niger* expression vectors pSZHG6R-man1R and pSZHG6R-man2R, respectively. A one-copy pSZHG6R-man1RTg vector with T*glaA* terminator was also generated. The double-digested vector (*Nhe* I/*Hin*d III) was further ligated to the double-digested (*Xba* I/*Hin*d III) two-copy expression vector to obtain the three-copy *A. niger* expression vector pSZHG6R-man3R containing a *glaA* site stimulating high *man* expression. The construction of the pSZHG6RP-man2R plasmid vector followed the same strategy as pSZHG6R-man2R, except that the *SglaA* signal peptide was replaced with the *SpepB* signal peptide. All constructs are shown in Figure 1.

**TABLE 1:** Primers used in this study.

Primers	Sequence (5′-3′)	Application
ManMC-FW	TCT​AGA​CTG​CCG​AAA​GCC​TCC​CCT	Clone man gene
ManMC-RW	AAG​CTT​AGA​TCT​TAA​GCA​CTA​CCA​ATA​GCA​GC	Clone man gene
glaA3p-FW	CGC​TAT​AGG​CTG​GTT​CTC​C	Transformant identification
glaA4p-RW	TCG​GCT​ATT​ATT​GAA​CTG​GG	Transformant identification
man-FW	CCG​GCG​AGA​CGG​ATT​TCT​AT	Real-time PCR identification
man-RW	ATC​GCA​ACT​CGG​ACA​TCT​CG	Real-time PCR identification
actA-FW	CCA​CGA​GAC​CAC​CTT​CAA​CTC​CA	Real-time PCR identification
actA-RW	CCA​CCG​ATC​CAG​ACG​GAG​TAC​TTG​C	Real-time PCR identification
bipA-FW	ACCAATACGCCGCCAACC	Real-time PCR identification
bipA-RW	CAG​CGT​GGG​TAA​CCT​TCT​TTC	Real-time PCR identification
pdiA-FW	AGC​TCA​AGG​CGA​AGA​ATA​TCC​CT	Real-time PCR identification
pdiA-RW	TAA​GGC​TTG​CTA​GAG​TCA​ACA​CC	Real-time PCR identification
hacA-FW	ACC​GAC​TAC​TCC​CCT​ACC​TTG	Real-time PCR identification
hacA-RW	AGC​CGA​CGT​CAA​AGA​GAG​AG	Real-time PCR identification
amyA-FW	GCC​CAT​CTA​CAA​AGA​CGA​CA	Real-time PCR identification
amyA-RW	ACA​TTT​CCA​TCC​GAA​CCA​AC	Real-time PCR identification

### 2.3 Generation of mannanase-expressing *A. niger* strains

*A. tumefaciens* ‘AGL1’ cells were freeze-thaw transformed with the pSZHG6R-man1R, pSZHG6R-man2R, pSZHG6RP-man2R, and pSZHG6R-man3R plasmids. The positive clones were utilized for the *A. tumefaciens*-mediated transformation of *A. niger.* Briefly, approximately 50 µL of homogenized *A. niger* ‘TH-2’ mycelium was mixed with 100 µL of pSZHG6R-man1R *Agrobacterium* solution, pSZHG6R-man2R *Agrobacterium* solution, or a pSZHG6RP-man2R/pSZHG6R-man3R *Agrobacterium* solution mixture in a 1.5 mL centrifuge tube and plated onto a cellophane-covered PDA plate containing 200 mmol·L^−1^ acetosyringone. Following 2 days of culture at 25 °C, the cellophane was inverted onto a screening plate containing 200 mmol·L^−1^ cephalosporin and 500 mmol·L^−1^ hygromycin. The cellophane was removed after 1 day and the plate was incubated for an additional 5–6 days at 30 °C until *A. niger* colonies were visible. Resistant colonies were selected and inoculated individually into PDA liquid medium containing 200 mmol·L^−1^ cephalosporin and 500 mmol·L^−1^ hygromycin for secondary screening. The co-culture and screening procedures were performed as described above. *Agrobacterium* transformants harboring pSZHG6R-man2R were used to genetically transform the *Vps10* knockout strain TH-2 (Vps::pyrG), while those carrying pSZHG6R-man3R were used to transform the *asAA* knockout strain TH-2 (asAA::pyrG).

Genomic DNA was extracted from *A. niger* hyphae exhibiting normal growth following the second round of screening. Genomic DNA extraction from *Aspergillus niger* was performed according to the manufacturer’s instructions for the Kangwei Century Plant Genomic DNA Extraction Kit (CW0553S). PCR was performed using the obtained genomic DNA and the homologous recombination primers glaA3p-FW and glaA4p-RW (Table 1). The host strain’s genomic DNA was used as a positive control and water was used as a negative control. Finally, the amplicons were evaluated using agarose gel electrophoresis. The presence of a DNA fragment identical in size to the positive control and no bands associated with the negative control indicated successful selection of a pure mannanase-producing *A. niger* strain.

### 2.4 Analysis of mannanase protein expression and enzyme activity

Fresh mycelia from the recipient and recombinant *A. niger* strains were cultured in PDA liquid medium for 3–4 days. Subsequently, the entire cultures were inoculated into 100 mL of liquid fermentation medium at a 10% (v/v) inoculum. Following 12 days of fermentation at 30 °C and 180 r·min^−1^, the supernatants were evaluated via polyacrylamide gel electrophoresis (CW0022M protein gel; 7 µL sample) carried out at 50 V using a vertical electrophoresis tank (Bio-Rad). The voltage was adjusted to 100 V once the samples reached the interface between the separating gel (bromophenol blue), and electrophoresis was continued until the bromophenol blue migrated to a distance of 1–2 cm from the bottom. The entire electrophoretic process was completed in approximately 1–2 h. Proteins were visualized via Coomassie Brilliant Blue R-250 (0.25%) staining either for 3–5 h or overnight. Stained gels were subsequently rinsed with washing solution and the decolorizing solution was replaced regularly until protein bands were clearly visible. Gray-scale analysis of protein bands was performed using ImageJ software. The AnMan protein expressed in the recipient strain was used as an internal loading control. The relative expression level of the target protein was determined by calculating the ratio of the integrated optical density of the target protein band to that of the corresponding internal control band. Normalization was subsequently performed using strain M2R as the reference sample, with all target protein relative expression levels expressed relative to that of M2R, enabling accurate inter-group comparisons and systematic data analysis.

To evaluate mannanase enzyme activity, approximately 0.5 g of konjac flour was dissolved by continuous stirring in 70 mL of buffer solution (0.05 mol·L^−1^ Na_2_HPO_4_·12H_2_O-C_6_H_8_O·7H_2_O; pH 3.5), and the final volume was adjusted to 100 mL with buffer to obtain the substrate. Next, 0.9 mL of the konjac flour substrate solution was added to both the sample and blank control tubes and incubated in a water bath at 70 °C for 3 min to establish equilibrium. Subsequently, 0.1 mL of the diluted enzyme solution was added to the sample tube and thoroughly mixed, while the blank control tube was left untreated. Both tubes were incubated at 70 °C for 10 min. Then, 3.0 mL of DNase was thoroughly mixed with the sample while 3.0 mL of dinitrosalicylic acid reagent and 0.1 mL of enzyme solution were mixed with the blank. Both tubes were then boiled in a water bath for 15 min, cooled down, and topped up with water to achieve a final volume of 15 mL. Finally, the absorbance (550 nm) of each mixture was measured and the enzyme activity was calculated. Three sets of samples and blank controls were maintained.

The enzyme activity was calculated using the following formula:

X=A×F×K×6÷0.1 (1)

where A denotes the absorbance value, F represents the enzyme dilution factor, K denotes the slope of the standard calibration curve, 0.1 is the volume of enzyme solution in mL, and 6 is the time conversion factor. Typically, one unit (U) of enzyme is defined as the quantity needed to hydrolyze 1.0 mg of reduced sugar (galactose) in konjac flour at a temperature of 70 °C and a pH of 3.5 per hour.

### 2.5 RNA isolation, cDNA synthesis, and real-time fluorescence quantitative PCR

After 5 days of shake flask fermentation culture, recombinant and host mycelial precipitate was harvested from the shake flasks and thoroughly ground with liquid nitrogen. An Ultrapure RNA Kit (CW0597S) was utilized for RNA extraction. RNA integrity was assessed using agarose gel electrophoresis, while RNA concentration and purity were determined using a Nanodrop spectrophotometer. The first-strand cDNA was synthesized using total RNA as the template and a HiFiScript gDNA Removal cDNA Synthesis Kit (CW2582M) with genomic DNA removal functionality. Real-time quantitative PCR (qPCR) was subsequently performed using cDNA as the template and actA as the internal reference gene, with a BIO-RAD real-time PCR system and UltraSYBR One-Step RT-qPCR Kit (CW2624S), to assess the transcription levels of *man*, *hacA*, *bipA*, and *pdiA* in the strain. The real-time quantitative PCR (qPCR) amplification protocol was as follows: initial pre-denaturation at 95 °C for 30 s, followed by 40 cycles of denaturation at 95 °C for 5 s and annealing/extension at 60 °C for 30 s. A subsequent melting curve analysis was performed with denaturation at 95 °C for 15 s, annealing/extension at 60 °C for 5 s, and a final denaturation at 95 °C for 15 s. The PCR reaction mixture consisted of the following components in a total volume of 25 µL: 12.5 µL of 2×MagicSYBR Mixture, 0.5 µL of forward primer, 0.5 µL of reverse primer, 1 µL of template cDNA, and 10.5 µL of nuclease-free water. Gene expression levels were quantified using the 2^−ΔΔC^_t_ method.

### 2.6 Morphological analysis of *A. niger* and quantification of hyphal biomass

Host and recombinant *A. niger* mycelia were screened onto solid PDA and incubated at 30 °C for 3–4 days until distinct dry colonies were visible. Subsequently, healthy fungal colonies (0.1 cm diameter) were sampled with a pipette, transferred onto solid fermentation medium, and incubated at 30 °C. Colony growth status was monitored and photographed on alternating days. Next, 10% (v/v) inocula of both host and recombinant mycelia were inoculated into 30 mL of liquid fermentation medium and incubated at 30 °C and 180 r·min^−1^ for 10 days. At the end of the incubation period, the fermented material was collected and centrifuged at 13,000 r·min^−1^ for 10 min. The process was repeated thrice, and the weight of the pooled mycelia was measured to quantify biomass accumulation.

### 2.7 Analysis of substrate specificity for mannanase

The diluted mannanase fermentation supernatant was separately added to substrates containing 0.5% konjac flour, 0.5% locust bean gum, 0.5% guar gum, and 0.5% sesbania gum, followed by thorough mixing. The resulting mixtures were then incubated at 70 °C for 2 h. Subsequently, the reaction systems were heated at 100 °C for 10 min to ensure complete inactivation of enzyme activity. The inactivated mixtures were centrifuged at 12,000 r·min^−1^ for 10 min and the resulting supernatants were carefully collected. The product components were identified and quantified using mass spectrometry (MS).

### 2.8 Thin-layer chromatography (TLC) methodology

The samples M, H, T, and G were first subjected to degreasing, followed by the preparation of appropriately sized cellulose thin-layer plates. Sample solutions were applied onto the thin-layer plates using capillary tubes. Following completion of spotting, the plates were transferred to a developing chamber to initiate the development process. The development process was terminated when the solvent front reached the upper edge of the thin-layer plate. The plate was then removed and immersed in the developing reagent (10% sulfuric acid in ethanol), followed by heating at 120 °C in an oven to visualize the developed bands. The reference standards used comprised a series of mannose oligosaccharides, including mannobiose, mannotriose, mannotetraose, mannopentaose, mannohexaose, and mannoheptaose.

### 2.9 Experiment on oligosaccharide mass spectrometry

Sample preparation: An appropriate amount of sample was dissolved in acetonitrile-water solution, followed by high-speed centrifugation. The supernatant was collected and filtered through a 0.22 μm microporous membrane prior to analysis. Mass spectrometric detection was carried out using an Agilent 6500 series quadrupole-time-of-flight tandem mass spectrometer (QTOF-6500).

Chromatographic analysis conditions: Separation was performed on an Acquity UPLC BEH Amide column (100 mm × 2.1 mm, 2.5 μm). Mobile phase A consisted of water containing 0.1% formic acid, and mobile phase B was acetonitrile. The elution program was as follows: 0–2 min, 70%–65% B; 2–14 min, 65%–55% B; 14–18 min, 55%–70% B; and 18–20 min, maintained at 70% B. The column temperature was set at 40 °C, and the injection volume was 2 μL. The mass spectrometry ion source was an electrospray ionization source (ESI), operated in positive ion mode. The mass scanning range was set from m/z 200 to 2000.

### 2.10 Analysis of the process conditions for preparing mannan oligosaccharides via mannanase-catalyzed hydrolysis of konjac flour

A series of single-factor experiments were conducted using reducing sugar yield as the evaluation index to systematically assess the preparation process of konjac mannan oligosaccharides. Single-factor experiments were conducted under the following conditions: reaction temperature (30, 40, 50, 60, 70, and 80 °C), pH value (2.5, 3.5, 4.5, 5.5, 6.5, and 7.5), enzyme dosage (0.05, 0.10, 0.15, 0.20, 0.25, 0.30, 0.35,0.40, 0.45, and 0.50 mg·mL^−1^), reaction time (1, 2, 3, 4, 5, 6, 7, 8, 9, and 10 h), and konjac powder concentration (5, 10, 15, 20, 25, and 30 g·L^−1^).

### 2.11 Data processing and analysis

The calculated data were subjected to one-way ANOVA using SPSS software to assess significant differences among groups. GraphPad Prism v8.0 was employed for data visualization. All experimental measurements were collected independently, with each group comprising at least three biological replicates. Results are presented as mean ± standard deviation (SD). Different lowercase letters denote statistically significant differences at P < 0.05.

## 3 Results

### 3.1 Establishment of an efficient mannanase-producing *A. niger* strain

Utilizing expression systems to generate recombinant proteins can address production challenges such as low protein yield, high cost, and difficulties associated with protein separation and purification. The key to enhancing recombinant protein yields lies in employing an appropriate, effective, and optimized strategy. Here, we first constructed four expression vectors containing different copy numbers of the *man* gene and different signal peptides via enzymatic ligation: pSZHG6R-man1R (one expression cassette and the *glaA* signal peptide), pSZHG6R-man2R (two expression cassettes and the *glaA* signal peptide), pSZHG6RP-man2R (two expression cassettes and the *pepB* signal peptide), and pSZHG6R-man3R (three expression cassettes and the *glaA* signal peptide). Prior to transformation into *A. tumefaciens* ‘AGL1’, double digestion with *Xba* I and *Hind* III was performed to confirm each expression vector (Supplementary Figure S1). Research suggests that gene sites exhibiting high expression also exhibit enhanced transcription efficiency, resulting in increased recombinant protein expression (Dong et al., 2019; Li et al., 2020; Kong et al., 2022). We incorporated *man* at the native high-expression locus of the endogenous *glaA* gene in the host strain through *Agrobacterium*-mediated transformation. Specifically, *A. tumefaciens* strains carrying the *AnMan* recombinant Ti plasmid were co-cultivated with *A. niger*. Finally, we isolated individual *A. niger* colonies and designated pure recombinant strains as follows: M1R (pSZHG6R-man1R), M2R (SglaA, glaA::man2R), M3R (SglaA, glaA::man3R), ΔM3R (SglaA, glaA::man3R, asAA::pyrG), ΔVM2R (SglaA, glaA::man2R, vps10::pyrG), and PM2R (SpepB, glaA::man2R). The strains used in this study and the characteristics of the constructed derivatives are summarized in Table 2.

**TABLE 2:** List of major strains.

Strains	Features	Source
*A. niger* ‘TH-2’	Receptor strain	This laboratory preserved
TH-2 (Vps::pyrG)	Receptor strain	This laboratory preserved
TH-2 (asAA::pyrG)	Receptor strain	This laboratory preserved
M1R	SglaA, glaA::man1R	This study constructed
M2R	SglaA, glaA::man2R	This study constructed
M3R	SglaA, glaA::man3R	This study constructed
ΔAM3R	SglaA, glaA::man3R, asAA::pyrG	This study constructed
ΔVM2R	SglaA, glaA::man2R, vps10::pyrG	This study constructed
PM2R	SpepB, glaA::man2R	This study constructed

### 3.2 Secretion and expression of mannanase in the recombinant *A. niger* strains

**FIGURE 2:**
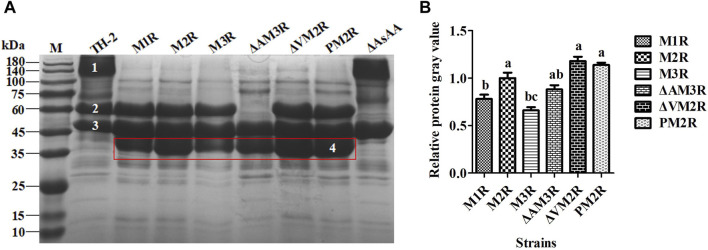
**(A)** SDS-PAGE analysis of secreted proteins from recombinant *Aspergillus niger* strains. M: molecular weight marker (prestained, 180 kDa). Identified bands: (1) glucoamylase (GlaA), (2) acid-stable α-amylase (AsAA), (3) α-amylase (AmyA), and (4) recombinant mannanase (AnMan). **(B)** Relative gray value analysis of SDS-PAGE bands quantifying recombinant mannanase expression.

The expression level and enzymatic activity of mannanase were further evaluated in the recombinant strain following 12 days of fermentation. The SDS-PAGE results are presented in Figure 2A. Lane 1 corresponds to glucoamylase (GlaA), lane 2 to acid-stable α-amylase (AsAA), lane 3 to α-amylase (AmyA), and lane 4 to recombinant mannanase (AnMan). Compared to the host strains TH-2 and ΔAsAA, all six recombinant strains exhibited distinct AnMan protein bands at approximately 37 kDa (Figure 2A; lane 4), and band intensity was positively correlated with protein expression level. Furthermore, no Gla protein expression was detected in lane 1, indicating that the GlaA gene had been successfully replaced by the AnMan expression cassette. All six strains were confirmed to be pure homologous recombinants. The protein band intensity of strain M2R was significantly higher than that of M1R and M3R, whereas M3R exhibited a downregulated expression pattern. These results suggest that double-copy integration may represent the optimal gene dosage, and a moderate increase in copy number can enhance recombinant protein expression. The protein band intensities of the strains ΔVM2R and PM2R, which were constructed on the basis of the double-copy, were both higher than that of M2R, indicating that either the knockout of the Vps10 gene or the replacement with the SpepB signal peptide could further promote the secretion expression of AnMan, with the effect of Vps10 deletion being more significant. The protein band intensity of strain ΔAM3R, constructed on a triple-copy background, was significantly higher than that of M3R, indicating that deletion of the high-secretion background protein AsAA at position 2 alleviates copy number-induced expression inhibition and thereby enhances AnMan production. To further quantify the differences in protein expression, strain M2R was used as the reference control for normalization. The gray values of AnMan protein bands in strains M1R, M3R, ΔAM3R, ΔVM2R, and PM2R were quantified using ImageJ software and normalized to the corresponding value of M2R (Figure 2B). The relative expression levels of AnMan protein in these strains were determined to be 0.78-, 0.66-, 0.88-, 1.18-, and 1.14-fold compared to M2R, respectively. Notably, mannanase expression was stronger in ΔVM2R and PM2R, but weaker in M1R and M3R, than M2R.

**FIGURE 3:**
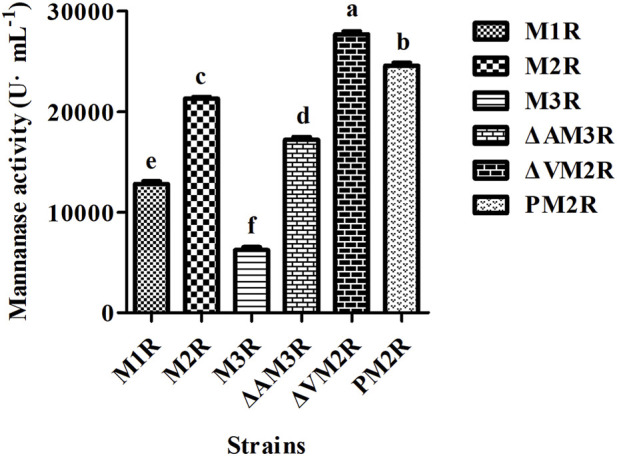
Enzymatic activity of mannanase in recombinant *Aspergillus niger* strains. Data are shown as the means ± standard deviations (SD) of three independent replicates. Different lowercase letters denote statistically significant differences among the strains (*P* < 0.05). When different groups do not share the same letter, this indicates significant differences between them.

Subsequent enzymatic activity assays performed after 12 days of fermentation revealed that the mannanase activities for M1R, M2R, M3R, ΔAM3R, ΔVM2R, and PM2R were 12805.92 U·L^−1^, 21289.84 U·L^−1^, 6242.89 U·L^−1^, 17207.96 U·L^−1^, 27692.80 U·L^−1^, and 24571.36 U·L^−1^, respectively (Figure 3). Relative enzyme activities were calculated with M2R as the reference strain, and the values for M1R, M2R, M3R, ΔAM3R, ΔVM2R, and PM2R were determined to be approximately 60.15%, 100%, 29.32%, 80.83%, 130.08%, and 115.41% of M2R activity, respectively. These results suggest that doubling the copy number of the gene expression cassette can significantly increase the expression and secretion of the mannanase protein. On the other hand, tripling the copy number resulted in a notable decrease in mannanase expression. It is likely that the excessive gene dosage led to misfolded protein aggregation in the ER, triggering the UPR response and secretion pressure, ultimately decreasing mannanase expression. This finding emphasizes the importance of controlling gene dosage to optimize protein expression (Wakai et al., 2019; Liu J. et al., 2020; Xia et al., 2021).

Furthermore, with the M3R strain as the reference, the relative enzymatic activity of ΔAM3R was 275.64%, which was significantly higher than that of M3R by 175.64%, suggesting that deletion of the background protein AsAA mitigated ER stress and bolstered appropriate protein folding. In addition, ΔVM2R exhibited 30.08% higher enzyme activity than M2R, indicating that deleting the degradation pathway-related gene *Vps10* impeded mannanase degradation while enhancing mannanase secretion. As a result, mannanase was effluxed into the extracellular space in ΔVM2R, leading to a substantial increase in recombinant protein expression. Finally, enzyme activity in the PM2R strain was 15.41% higher than that in the M2R strain. This observation suggests that the S*pepB* signal peptide efficiently regulates mannanase expression and secretion by facilitating proper targeting of the protein to the ER.

Compared with the highest enzyme activity (11,600 U·L^−1^) achieved by the thermophilic and acidophilic β-mannanase (ManAK) from *Aspergillus kawachii* expressed in *Pichia pastoris* (Liu Z. et al., 2020), and the enzyme activity (22,100 U·L^−1^) obtained by expressing the β-mannanase (AnMan26) from *Aspergillus niger* in *Pichia pastoris* (Wang et al., 2021), the β-mannanase produced by *Aspergillus niger* in this study exhibited a higher enzyme activity level under shake flask fermentation conditions, demonstrating superior enzyme production performance. In addition, the β-mannanase (mRmMan5A) from *Rhizomucor miehei* was heterologously expressed in *Saccharomyces cerevisiae*, and under high-density fermentation conditions, the expression level reached 79,680 U·mL^−1^ (Li et al., 2018). In contrast, this study achieved a high enzyme yield under conventional shake flask fermentation conditions, demonstrating the strong enzymatic production potential of the strain. With further optimization of fermentation parameters and scale-up cultivation in bioreactors, the enzyme yield is expected to be significantly enhanced.

### 3.3 The impact optimization on the transcriptional activity of the recombinant *A. niger* strains

**FIGURE 4:**
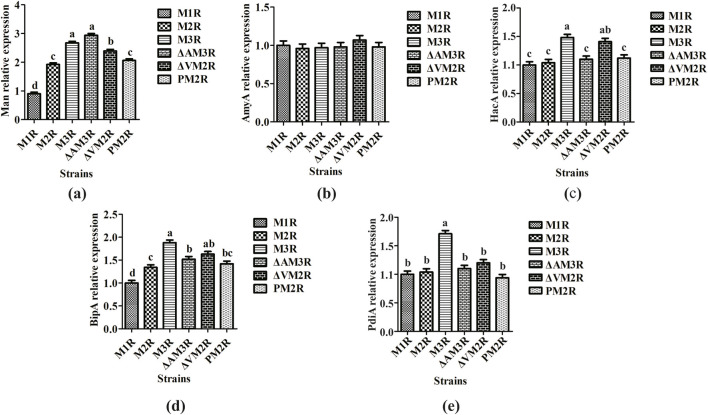
Transciptional analysis of recombinant *Aspergillus niger* strains. RT-qPCR quantification of transcripts levels for: **(a)** *man*, **(b)** *amyA*, **(c)** *hacA*, **(d)** *bipA*, and **(e)** *pdiA.* Data are shown as the means ± standard deviations (SD) of three independent replicates. Different lowercase letters denote statistically significant differences among the strains (*P* < 0.05). When different groups do not share the same letter, this indicates significant differences between them.

Because significant variation was observed in mannanase expression among the recombinant strains, we utilized RT-qPCR to investigate whether these differences were associated with the transcription of genes related to ER stress and the UPR. Specifically, we quantified transcript levels of the target gene *man*, the endogenous high-expression gene *amyA*, and the UPR indicator genes *hacA*, *bipA*, and *pdiA*. Relative to M1R, the transcription levels of the man gene in the M2R, M3R, ΔAM3R, ΔVM2R, and PM2R strains were approximately 192%, 267%, 294%, 239%, and 206%, respectively (Figure 4a). Analysis of gene copy numbers revealed that M1R, M2R, M3R, ΔAM3R, ΔVM2R, and PM2R harbored 1, 2, 3, 3, 2, and 2 integrated copies of the man gene, respectively. When normalized to M3R as the reference strain, the relative transcription level of man in ΔAM3R reached 110.11% of the control level, representing a 10.11% increase compared to M3R. When normalized to M2R as the reference strain, the relative transcription levels of man in ΔVM2R and PM2R were 124.48% and 107.29% of the control level, respectively, representing increases of 24.48% and 7.29% compared to M2R. It has been well established that gene transcription levels are positively correlated with mRNA stability (Yuan et al., 2020). The modest transcriptional upregulation of man may be attributed to enhanced mRNA stability conferred by the knockout of background proteins, loss of Vps10, and replacement of the SpepB signal peptide. Finally, no significant differences in *amyA* transcription were observed among strains (Figure 4b), indicating a high degree of consistency in the fermentation conditions applied to each strain.

We also evaluated differences in the expression levels of the UPR-related genes *hacA*, *bipA*, and *pdiA* among strains (Figures 4c–e). These UPR genes were upregulated in each of the strains compared to M1R, although a statistically significant difference was observed only between M1R and M3R. It may be inferred that M3R experienced pronounced ER stress and folding defects, necessitating greater assistance from molecular chaperones and folding enzymes to ensure appropriate protein folding. These findings collectively suggest that the excessive accumulation of exogenous proteins following upregulated expression likely results in improper folding and inefficient secretion, thereby reducing protein expression overall. Moreover, protein overexpression challenges the ER, triggering the UPR and ERAD to alleviate ER stress (Baker, 2018; Sun and Su, 2019).

### 3.4 The impact optimization on the growth status of the recombinant *A. niger* strains

**FIGURE 5:**
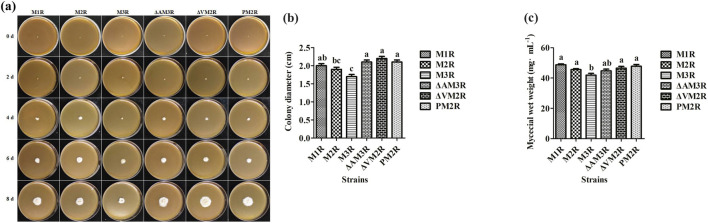
Growth profiles of recombinant *Aspergillus niger* strains. **(a)** Colony morphology from day 0 to day 8 on solid fermentation medium. **(b)** Average colony diameter measured on day 10. **(c)** Mycelial wet weight following 12 days of fermentation on solid medium.

Typically, the mycelial growth status of a particular fungal strain demonstrates the efficacy of various optimization strategies and serves as a crucial metric for assessing the efficiency of protein secretion. After inoculating recombinant *A. niger* into fermentation medium, growth was evaluated after 0, 2, 4, 6, and 8 days (Figure 5a). In addition, the average colony diameter was measured on day 8 (Figure 5b) and the wet hyphal weight was measured on day 12 (Figure 5c). Compared to M1R, no significant differences in colony size or morphology were observed among ΔAM3R, ΔVM2R, or PM2R. It appears that deleting the background protein AsAA, deleting the protein degradation pathway-related gene *Vps10*, and utilizing high-efficiency signal peptide S*pepB* have a minimal impact on hyphal growth. On the other hand, M3R exhibited reduced growth compared to M1R, consistent with the protein secretion results (Figure 5c). Together, these results suggest that M3R experienced considerable ER pressure, negatively impacting hyphal growth.

### 3.5 Process analysis of mannanase-mediated synthesis of mannooligosaccharides

**FIGURE 6:**
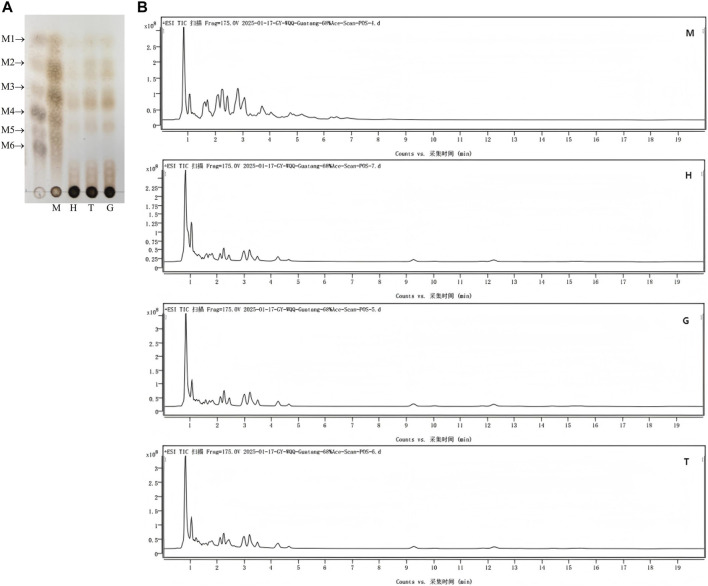
Component analysis of oligosaccharides released after 2-h enzymatic hydrolysis of different mannans using mannanase. **(A)** Thin-layer chromatography (TLC) of hydrolysates. M1-M6: standard oligosaccharides ranging from mono- to hexasaccharides. **(B)** Total ion chromatograms of hydrolysis products from konjac flour (M), locust bean gum (H), sesbania gum (T), and guar gum (G).

The chromatographic and MS analyses of oligosaccharide products resulting from the enzymatic hydrolysis of konjac flour, locust bean gum, guar gum, and sesbania gum incubated with mannanase for 2 h are shown in Figures 6A,B, respectively. Correlations between mannan oligosaccharides and their corresponding m/z values obtained via MS are summarized in Table 3. Based on the molecular weight analysis, it can be inferred that all four polysaccharide substrates yielded mannan oligosaccharides ranging from monosaccharides to nonasaccharides following enzymatic hydrolysis. Konjac flour produced primarily monosaccharides to heptasaccharides, the relative abundances of mannosobiose, mannosotriose, mannosotetraose, and mannosopentaose were notably high, reaching 25.65%, 27.53%, 18.78%, and 16.31%, respectively. The levels of mannotriose and mannohexose were relatively low, at 6.20% and 5.53%, respectively, while octasaccharide and nonasaccharide were not detected. The total content of disaccharides and higher-degree oligosaccharides reached 94.47%, indicating the efficient production of mannan oligosaccharides in this system. These results demonstrate that the mannanase exhibits significant potential for the preparation of oligosaccharide-based products. In contrast, pentasaccharides and hexasaccharides were absent in the hydrolysis products of locust bean gum, guar gum, and sesbania gum.

**TABLE 3:** Oligosaccharide composition.

Oligosaccharide	Monosaccharide	Disaccharide	Trisaccharide	Tetrasaccharide	Pentose	Hexose	Heptose	Octose	Nonasaccharides
m/z	203.0528	365.105	527.158	689.211	829.282	991.335	1175.370	1337.422	1499.474

**FIGURE 7:**
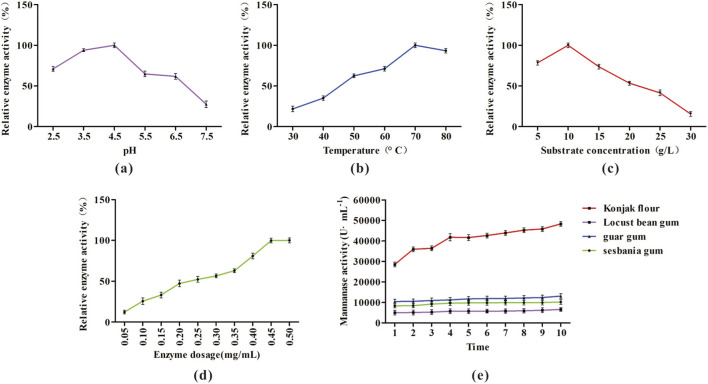
Optimization of mannan oligosaccharide (MOS) production from konjac flour using recombinant mannanase. **(a)** Effect of pH. **(b)** Effect of reaction temperature. **(c)** Effect of konjac flour concentration. **(d)** Effect of enzyme dosage. **(e)** Effect of reaction time. Each parameter was systematically varied to determine optimal conditions for MOS yield.

To further optimize the enzymatic synthesis of oligomannose using mannanase, konjac flour was selected as the model substrate and a series of systematic experiments were carried out. As shown in Figure 7, maximum oligomannose production was achieved at a pH of 4.5 (Figure 7a), a temperature of 70 °C (Figure 7b), a konjac flour concentration of 10 g·L^−1^ (Figure 7c), and a mannanase enzyme dosage of 0.5 mg·mL^−1^ (Figure 7d). Furthermore, the content of reducing sugars generated steadily increased with prolonged reaction time (Figure 7e).

## 4 Discussion

In this study, we sought to evaluate the effects of optimizing the gene expression cassette copy number, eliminating the background protein AsAA, deleting the degradation pathway-related gene Vps10, and utilizing different signal peptides (SglaA and SpepB) on mannanase expression in *A. niger*. The M1R strain was used as the control, the recombinant strain exhibited a maximum relative mannanase activity that increased by 216.25% (27,692.80 U·L^−1^), surpassing the reported activities of the thermophilic and acidophilic β-mannanase ManAK expressed in the *P. pastoris* system (Liu Z. et al., 2020) and AnMan26 from *A. niger* (Wang et al., 2021). Furthermore, the expression level demonstrated competitive performance compared to similar studies in the field (Nath and Kango, 2024). Currently, the recombinant expression systems for mannanase primarily encompass prokaryotic systems (exemplified by *E. coli*), eukaryotic systems-including yeast and *filamentous fungi*-and plant-based expression platforms, as summarized in Table 4. Compared to yeast-based expression systems, *filamentous fungal* systems offer the advantage of highly efficient expression with minimal process complexity, making them better suited for rapid strain screening and small- to medium-scale production during the early stages of industrial development. The application of CRISPR-Cas9 gene editing technology has substantially shortened the genetic manipulation cycle, establishing *filamentous fungal* systems as a leading platform for current industrial applications. Although the *E. coli* expression system enables high-level protein production (Zhou et al., 2018), the majority of recombinant enzymes are prone to forming inclusion bodies and lack essential glycosylation modifications, leading to low levels of soluble enzymatic activity. In recent years, the lettuce chloroplast system (Fatima et al., 2025) has demonstrated the potential for “purification-free” production-particularly advantageous for oral enzyme formulations and a high large-scale production capacity of up to 9 × 10^9^ U/acre/year. However, this system depends on agricultural cultivation, which entails a prolonged growth cycle, and is prone to contamination with plant-derived polysaccharides. These impurities compromise the purity of MOS, thereby limiting its ability to match the process simplicity of the *A. niger* system, in which MOS purity exceeding 90% can be achieved through simple filtration following enzymatic hydrolysis. Therefore, plant-based expression systems are better suited for the development of oral enzyme preparations, whereas the *A. niger* system offers greater advantages in the industrial-scale production of MOS. *A. niger* exhibits highly efficient secretory expression, enabling recombinant enzymes to be directly secreted into the fermentation broth. Following enzymatic hydrolysis of the substrate, effective separation of enzymes from MOS can be achieved through conventional filtration, eliminating the need for complex purification procedures. This feature aligns closely with the commercial production platforms employed by leading enzyme manufacturers such as Novozymes and Danisco, where over 80% of their mannanase production capacity is based on *filamentous fungal* systems. In addition, the enzymatic properties of this recombinant enzyme are highly aligned with industrial application requirements. It exhibits an optimal reaction temperature of 70 °C and maintains excellent stability within the range of 30 °C–70 °C, retaining full enzymatic activity after incubation at 70 °C for 90 min. The optimal pH is 4.5, with stable catalytic activity observed between pH 3.5 and 6.5, indicating robust tolerance to both high-temperature sterilization and acidic conditions encountered in food processing.

**TABLE 4:** Comparative analysis of key characteristics of recombinantly expressed mannanase across different expression systems.

Expression system	Enzyme activity of mannanase	Cultivation cycle (h/d)	Key advantages	Key limitations	Optimal application scenarios
*E. coli*	6,041 U·mL^−1^	8–24 h	Genetic manipulation is efficient and cost-effective	It is prone to form inclusion bodies (refolding efficiency <30%); has weak secretion ability (accumulates intracellularly, complex purification); contains endotoxin, and the cost of removal accounts for 30%–50%.	Low-cost industrial enzyme preparations
*P*. *pastoris*	11,600 U·mL^−1^	24–72 h	Highly efficient secretion, excellent genetic stability, and robust post-translational modification; GRAS-certified strain.	Excessive glycosylation (100–200 mannose residues); methanol induction poses safety risks. The cost of methanol-compatible explosion-proof equipment is 20%–30% higher compared to standard systems.	Industrial enzymes
*A*. *niger*	10,028 U/gds	3–7 days	Strong extracellular secretion; post-translational modifications similar to higher eukaryotes; low cultivation cost; GRAS-certified strain.	Genetic manipulation is challenging due to low homologous recombination efficiency.	Large-scale industrial enzymes (the mainstream option)
*Plant*	8.64 × 10^9^ U per acre per year	months to years	Low production cost, suitable for large-scale production; post-translational modifications resemble those in mammals; high safety.	Low expression; long production cycle; environmentally sensitive genetic stability; challenging purification.	Pharmaceutical-grade enzyme preparations, *etc.*

Protein quantification, enzymatic activity assays, RT-qPCR analyses, and fungal growth experiments revealed that doubling the copy number of the gene expression cassette significantly increased mannanase expression while tripling the copy number reduced mannanase expression due to activation of the UPR response and reduced hyphal growth. This indicates that once the gene dosage surpasses a certain threshold, it results in extensive accumulation of unfolded or misfolded proteins in the ER, thereby negatively impacting cellular function. In the strain harboring three copies of the expression cassette and deficient in the highly secreted background protein AsAA, the expression level of mannanase AnMan was increased by 2.75-fold. This observation is consistent with the increased protein levels detected by SDS-PAGE, whereas RT-qPCR analysis revealed no significant change in transcript abundance, indicating that the upregulation of the target protein expression occurs primarily at the post-transcriptional level. Concomitantly, the transcription levels of the core UPR-related factors *hacA*, *bipA*, and *pdiA* were significantly downregulated in this strain (Figure 4), with no adverse effect on hyphal growth. Given that *hacA* is the central transcriptional regulator of the UPR pathway, its splicing-mediated activation triggers the expression of downstream molecular chaperone genes such as *bipA* and *pdiA* in response to the accumulation of unfolded or misfolded proteins in the ER. Thus, the downregulation of these genes’ transcript levels suggests a substantial alleviation of cellular ER folding stress. This finding indirectly suggests that AsAA, as a highly abundant secreted protein, contributes substantially to the endoplasmic reticulum’s folding burden; its deletion therefore reduces the load of non-essential proteins in the ER and alleviates resource competition within molecular chaperone networks and post-translational modification systems. Furthermore, the amelioration of the endoplasmic reticulum’s folding environment may enhance the proper folding efficiency of AnMan, minimize its degradation due to misfolding, and consequently facilitate its stable accumulation and efficient secretion. The strategy of knocking out non-target proteins to improve recombinant protein yield has been previously documented in *Aspergillus* systems (Gou et al., 2025). These studies collectively demonstrate that the genetic elimination of highly secreted, non-essential background proteins enables a more efficient reallocation of cellular resources to target protein synthesis and alleviates bottlenecks in protein folding and trafficking within the secretory pathway, thereby constituting a key strategy for optimizing recombinant protein expression in *filamentous fungi*. In the double-copy system, deletion of Vps10 resulted in a 1.3-fold increase in enzyme activity in the ΔVM2R strain relative to M2R. This enhancement is likely attributable to the disruption of the protein sorting pathway caused by the loss of Vps10. Previous studies have demonstrated that Vps10 functions as a vacuolar sorting receptor with a well-established role in mediating the targeted trafficking of cargo proteins to the vacuole (Luo et al., 2022). Based on these findings, we hypothesize that the loss of Vps10 abolishes its function in mediating the vacuolar sorting of AnMan, thereby redirecting the protein from the “vacuolar degradation/retention pathway” to the “secretory vesicle pathway”. This hypothesis is consistent with the observed phenotype of elevated extracellular AnMan secretion in the ΔVps10 strain. However, the magnitude of this increase was lower than that observed upon AsAA gene knockout, likely because only a minor fraction of mannanase is constitutively directed to the vacuole under wild-type conditions. This study has not directly assessed the distribution of AnMan in subcellular compartments such as vesicles and vacuoles by Western blotting (WB), and therefore lacks experimental validation of its subcellular localization, representing a critical gap in the current mechanistic interpretation. Future studies should prioritize the integration of techniques such as Western blotting (WB), co-immunoprecipitation (Co-IP), and shotgun LC-MS/MS to systematically characterize the subcellular distribution of AnMan and its interaction with Vps10, thereby enabling direct validation of whether Vps10 regulates extracellular AnMan secretion through modulation of vesicle sorting.

Furthermore, the impact of the SglaA signal peptide and SpepB signal peptide on the secretory expression of mannanase was investigated using two copies of the gene expression cassette. Employing the SpepB signal peptide resulted in a 1.15-fold increase in secretion compared to SglaA. The amino acid sequence and structural features of signal peptides enable the efficient targeting and translocation of target proteins to specific organelles or the extracellular environment, thus facilitating high-level secretory expression. This improvement may be attributed to the higher hydrophobic amino acid content in the SpepB signal peptide (65%) compared to that of SglaA (60%), as well as its β-sheet conformation, which contributes to the maintenance of AnMan’s overall structural stability. This enhanced stability facilitates transmembrane transport and extracellular secretion, thereby supporting efficient secretory expression. However, the enhancement conferred by the signal peptide was lower than that achieved through deletion of AsAA or Vps10, likely due to the relatively minor structural differences between the two signal peptides, which may have resulted in only a limited improvement in the expression level and enzymatic activity of the mature AnMan protein. In contrast, deletion of AsAA or Vps10 may have substantially altered the overall biosynthetic pathway and folding trajectory of AnMan, thereby triggering intracellular compensatory mechanisms or novel regulatory pathways. This enables more efficient production of biologically active AnMan, leading to a significantly enhanced expression outcome.

Compared with yeast expression systems, the *A. niger* platform offers superior extracellular protein secretion capacity, enhanced biosafety, and the ability to perform a broad range of complex post-translational modifications. In some cases, these modifications more closely resemble those of higher eukaryotic proteins. Notably, specific glycosylation patterns can markedly affect catalytic activity, thermal stability, and substrate specificity, thereby facilitating the production of recombinant enzymes with improved functional integrity and performance. In this study, mannanase expressed in *A. niger* exhibited relatively high enzyme activity compared with certain yeast-based systems. However, relative to state-of-the-art production levels both domestically and internationally, substantial room for improvement remains.

Mannan derived from different sources exhibits variation in both structure and degree of polymerization, both of which directly influence the hydrolytic efficiency of mannanase. Here, the highest hydrolytic efficacy was observed when incubating mannanase with konjac flour, with the primary hydrolysis products being manno-oligosaccharides such as mannobiose and mannotriose. Through systematic optimization of process parameters, the key factors affecting hydrolysis efficiency were identified and analyzed. Overall, optimal oligomannose production was achieved at a pH of 4.5, a temperature of 70 °C, a konjac flour concentration of 10 g·L^−1^, and a mannanase enzyme dosage of 0.5 mg·mL^−1^. The results of these single-factor experiments represent only preliminary optimizations. While they enable independent assessment of the influence of individual factors on final product yield, they are unable to capture the dynamic behavior of the mannanase hydrolysis reaction under the synergistic effects of multiple variables. Consequently, such an approach limits a comprehensive understanding of the hydrolysis mechanism and hinders the systematic optimization of process parameters. To achieve more rigorous and reliable process optimization, the response surface methodology (RSM) will be employed to systematically analyze the interactive effects of multiple variables and identify globally optimal conditions. In the large-scale production of MOS from konjac flour, the stability and catalytic activity of mannanase are highly susceptible to various process-related factors. Prolonged reaction durations may result in progressive enzyme inactivation, thereby compromising both the yield and quality of the final product. The implementation of enzyme immobilization technology offers a promising strategy to enhance operational stability, facilitate repeated enzyme use, reduce unit production costs, and improve overall process efficiency and economic viability.

## 5 Conclusion

This study demonstrates that increasing the copy number of the gene expression cassette, knocking out the background protein AsAA, deleting the degradation pathway-associated gene Vps10, and employing the SpepB signal peptide collectively lead to a significant enhancement in the secretion and expression levels of mannanase in *Aspergillus niger*, concomitantly restoring or improving the mycelial growth phenotype. Transcriptional analysis of the target gene man and unfolded protein response (UPR)-related genes reveals that the folding, trafficking, and degradation of secretory proteins in the endoplasmic reticulum collectively represent a major bottleneck limiting efficient recombinant enzyme expression in this host system. The high hydrolytic activity of the recombinant enzyme toward konjac powder provides direct evidence supporting its potential for industrial application. Based on these findings, this study has established a scalable engineering framework for industrial enzyme expression, providing a robust theoretical foundation and technical platform for the low-cost, large-scale production of MOS and the efficient biosynthesis of other industrial enzymes.

## Data Availability

The original contributions presented in the study are publicly available. This data can be found here: 10.5281/zenodo.18321073.
