# Supplementary material for: Optimization of mannanase expression in Aspergillus niger for enhanced production of mannan oligosaccharides
Source: Front Bioeng Biotechnol. 2026 Jan 30;13:1716075. doi: 10.3389/fbioe.2025.1716075 (PMC12901352; doi:10.3389/fbioe.2025.1716075)
Supplement: Supplementary file 1 [file DataSheet1.pdf]

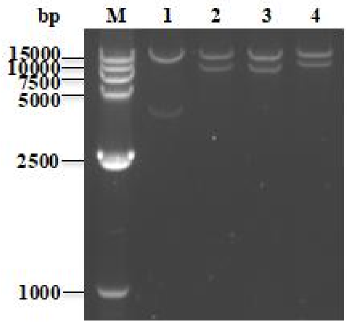

Supplementary Fig. S1 The enzymatic digestion results of the mannanase expression vector

Note：M-DL 15 000 bp；1-pSZHG6R-man1R（*Xba* I/*Hin*d III）; 2-pSZHG6R-man2R（*Xba* I/ *Hin*d III）; 3-pSZHG6RP-man2R（*Xba* I/*Hin*d III）; 4-pSZHG6R-man3R（*Xba* I/ *Hin*d III）
